# Analgesic combinations to reduce the stress/pain responses in ewes submitted to non-surgical embryo recovery: butorphanol associated with meloxicam or with dipyrone/hyoscine

**DOI:** 10.1590/1984-3143-AR2025-0177

**Published:** 2026-08-10

**Authors:** Ana Paula Pereira Schmidt, Ana Clara Sarzedas Ribeiro, Juliana Dantas Rodrigues Santos, Pedro Henrique Nicolau Pinto, Aline Matos Arrais, Joanna Maria Gonçalves Souza-Fabjan, Mário Felipe Alvarez Balaro, Jeferson Ferreira da Fonseca, Rodolfo Ungerfeld, Felipe Zandonadi Brandão

**Affiliations:** 1 Faculdade de Veterinária, Universidade Federal Fluminense – UFF, Niterói, RJ, Brasil; 2 Embrapa Caprinos e Ovinos, Coronel Pacheco, MG, Brasil; 3 Facultad de Veterinaria, Universidad de la República, Montevideo, Uruguai

**Keywords:** analgesia, animal welfare, sheep, transcervical embryo collection

## Abstract

The aim of this study was to compare the effectiveness of different analgesic protocols in reducing physiological stress markers in Santa Inês ewes undergoing non-surgical embryo recovery (NSER). In Experiment 1 (n = 32), ewes received a combination of butorphanol and meloxicam, whereas in Experiment 2 (n = 34), animals were treated with butorphanol combined with dipyrone and hyoscine. In Experiment 1, ewes received butorphanol (0.1 mg/kg IM) associated with meloxicam (1 mg/kg IV). In Experiment 2, animals were treated with butorphanol (0.1 mg/kg IM) combined with a dipyrone-hyoscine mixture (5 g dipyrone + 0.04 g hyoscine; 5 mL IM and 5 mL IV). Controls received equivalent volumes of saline. In Experiment 2, dipyrone-hyoscine was re-administered 24 h post-procedure. Ewes were synchronized, superovulated, and subjected to cervical dilation before NSER. Heart rate, respiratory rate, and blood biomarkers were recorded before sedation (BS), after sedation (AS), before cervical transposition (BCT), immediately after collection (IAC), and at 0.5, 1.5, 3, 6, 12, 24, and 48 h after collection (AC). In Experiment 1, respiratory rate was affected by treatment over time, it was greater (P=0.048) in control ewes than in ewes that received butorphanol associated with meloxicam at AS and 0.5hAC. No other variable differed significantly between groups. In Experiment 2, there was an interaction between treatment and time in heart rate. Heart rate was greater in ewes treated with butorphanol combined with a dipyrone-hyoscine mixture than in control ewes (P = 0.001), and there was also a significant interaction between treatment and time (P = 0.001). It was greater in treated ewes than in control ewes at AS, IAC, 0.5hAC, and 1.5hAC. No significant effects were detected for the remaining parameters. Under the tested conditions, neither analgesic protocol significantly reduced physiological indicators of stress.

## Introduction

Assisted reproductive technologies (ART) have become essential tools for accelerating genetic improvement in small ruminants. Among these, non-surgical embryo recovery (NSER) has been developed as a less invasive alternative to laparotomy, offering faster recovery and the possibility of repeated collections in donor ewes. Despite these advantages, NSER still induces pain and discomfort due to cervical manipulation and uterine distension (Santos et al., 2020; Ribeiro et al., 2023). Therefore, improving peri-procedural pain management is essential to ensure animal welfare and compliance with ethical standards.

The use of analgesic and anti-inflammatory drugs can minimize these adverse effects, but it is crucial to select combinations that effectively reduce stress and pain responses without compromising reproductive outcomes or embryo quality. Ribeiro et al. (2023) reported that meloxicam, a non-steroidal anti-inflammatory drug (NSAID) that inhibits prostaglandin synthesis, either alone or in combination with dipyrone, showed limited effectiveness in mitigating physiological stress responses during NSER. These findings highlight the need to explore alternative or complementary pharmacological strategies to optimize animal comfort during reproductive procedures.

Butorphanol, a synthetic opioid with central analgesic activity, and meloxicam are both widely used in veterinary medicine to manage perioperative pain and inflammation. When combined with dipyrone, hyoscine (also known as scopolamine) exerts antispasmodic, analgesic, and antipyretic effects, making this combination effective for managing visceral and colic pain in animals (Barros and Stasi, 2012; Spinosa et al., 2017). However, data on the impact of these drugs—alone or in combination—on reproductive tract function and stress modulation in small ruminants remain limited. Notably, butorphanol administration reduces stress responses associated with electroejaculation in rams (Ungerfeld et al., 2022). Yet, no studies have examined its effects during manipulation of the female reproductive tract.

Given this background, the aim of the present study was to compare two analgesic protocols combining butorphanol with (1) meloxicam (Experiment 1) or (2) dipyrone and hyoscine (Experiment 2) in reducing stress-related physiological responses during NSER in Santa Inês ewes. We hypothesized that both combinations would attenuate increases in heart rate, respiratory rate, cortisol, and glucose concentrations induced by the procedure, without adversely affecting embryo recovery outcomes.

## Methods

### Experimental location, animals, and experimental design

All procedures followed institutional and national guidelines for animal care and use in research and the Animal Use Ethics Committee of the Universidade Federal Fluminense approved the study (CEUA-UFF; Protocol No. 4783220822). The study was conducted during the winter season (July-August) at the Experimental Research Unit for Goats and Sheep, Universidade Federal Fluminense (22°S, 42°W), Cachoeiras de Macacu, Rio de Janeiro, Brazil.

Multiparous Santa Inês ewes, clinically healthy and free of reproductive disorders, were selected based on gynecological and ultrasonographic examinations. Animals were housed in individual raised pens with slatted floors under natural photoperiod and ambient temperature. The diet consisted of chopped elephant grass (*Pennisetum purpureum*), 300 g of a concentrate ration (16% crude protein) per ewe per day, and ad libitum access to water and mineralized salt (Salminas Ovinos, Nutriplan, Juiz de Fora, Brazil).

The study comprised two experiments, each designed to evaluate a different multimodal analgesic protocol for pain control during NSER. In Experiment 1, 32 ewes were randomly assigned to one of two groups: a treatment group (MBG; *n* = 16) receiving butorphanol and meloxicam, and a control group (CONG1; *n* = 16) receiving saline. In Experiment 2, 20 ewes were used in a crossover arrangement, in which all animals received both the treatment (BBG; butorphanol + dipyrone and hyoscine) and the control (CONG2; saline) protocols, with ten animals per sequence. A washout period of 119 days—equivalent to more than three complete estrous cycles in Santa Inês ewes—was applied to eliminate potential residual or carryover effects between treatments.

Due to logistical constraints, each experiment was conducted in four consecutive subgroups (four to six animals per subgroup). On each day, one subgroup underwent all procedures, and animals were allocated to treatment or control groups according to body weight to ensure balanced distribution across experimental conditions. Although the number of animals per group was limited by facility capacity and ethical considerations, the sample size was comparable to those in previous studies evaluating physiological and hormonal responses to analgesic protocols in sheep (Ribeiro et al., 2023; Balistrieri et al., 2023).

### Hormonal protocols

The estrous cycles were synchronized according to Balaro et al. (2016). On Day –6, intravaginal sponges containing 60 mg medroxyprogesterone acetate (Progespon; Syntex, Buenos Aires, Argentina) were inserted and remained in situ for six days. One day before sponge removal, ewes received 300 IU equine chorionic gonadotropin (eCG; Novormon, Syntex) and 0.24 mg sodium cloprostenol (Estron; Agener União Saúde Animal, São Paulo, Brazil) intramuscularly (IM). Thirty-six hours after sponge removal, lecirelin (25 µg; TEC-Relin, Agener União Saúde Animal) was administered IM.

Superovulation began 80 h after sponge removal (Day 0). A total of 133 IU porcine follicle-stimulating hormone (pFSH; Pluset, Biogénesis Bagó, Curitiba, Brazil) was administered IM in six decreasing doses (25%, 25%, 15%, 15%, 10%, 10%) at 12-h intervals (Ribeiro et al., 2025). The first dose coincided with the insertion of a new intravaginal progesterone implant (0.36 g; Primer PR, Agener União Saúde Animal), which remained until the fifth dose. The last pFSH dose was accompanied by 0.24 mg cloprostenol IM, followed 24 h later by lecirelin (25 µg IM).

For NSER, cervical dilation was induced according to Leite et al. (2018). Estradiol benzoate (100 µg; RIC-BE, Agener União Saúde Animal) diluted in 2.5 mL absolute ethanol and 2.5 mL saline was administered intravenously (IV), together with sodium cloprostenol (0.12 mg IM), 12 h before embryo collection. Additionally, oxytocin (100 IU IV; Ocitocina Forte UCB, Centrovet, Goiânia, Brazil) was given 15 min before NSER to facilitate cervical transposition.

### Pain control protocols and NSER

Before NSER, ewes were sedated with acepromazine (0.1 mg/kg IV; Acepran, Vetnil, Louveira, Brazil) and diazepam (0.2 mg/kg IV; Diazepam, Teuto, Anápolis, Brazil), followed by epidural anesthesia using ketamine hydrochloride (2.0 mg/kg IV; Cetamin, Syntec, Barueri, Brazil).

**Figure 1:**
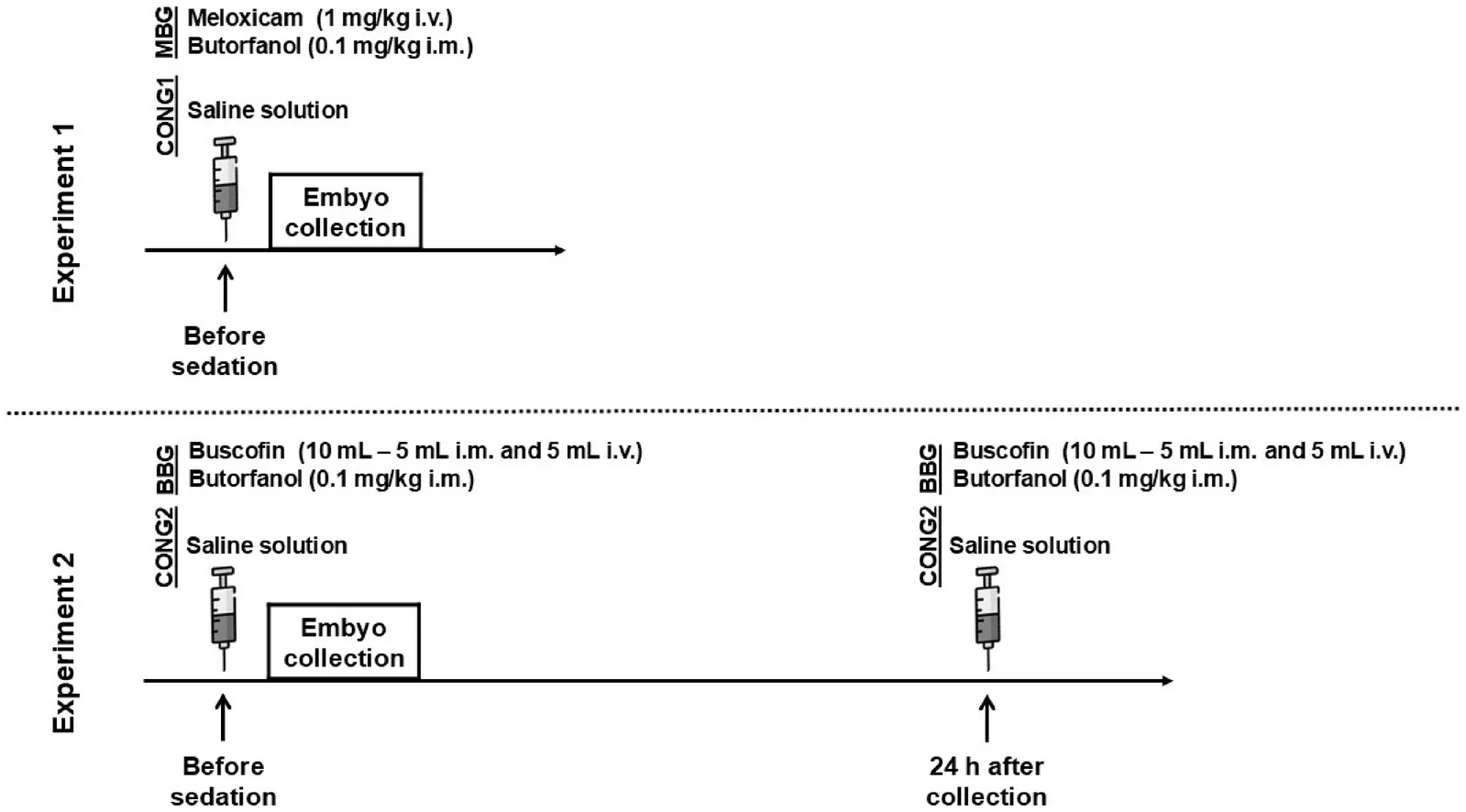
Schematic representation of analgesic protocols in Santa Inês ewes subjected to non-surgical embryo recovery.

In Experiment 1, MBG ewes received butorphanol (0.1 mg/kg IM; Butorfin 1%, Vetnil) combined with meloxicam (1 mg/kg IV; Maxicam 2%, Ourofino) at the time of sedation, immediately before NSER. In Experiment 2, BBG-treated ewes received butorphanol (0.1 mg/kg IM) combined with a commercial formulation containing dipyrone (5 g) and hyoscine (0.04 g) (Buscofin Composto, Agener União Saúde Animal) at a fixed total dose of 10 mL per ewe (5 mL IM + 5 mL IV). The same dose was re-administered 24 h after embryo collection. Control groups in both experiments received equivalent volumes of sterile saline through the same routes and at the same time points. Figure 1 shows the timeline of drug administration. For embryo collection, the cervix was fixed and tractioned, followed by uterine horn flushing and embryo recovery using a closed system (Circuito Embrapa for goat/sheep embryo recovery, Embrapa, Brasília, Brazil), as described by Fonseca et al. (2013).

### Stress and pain markers

Heart rate (HR) and respiratory rate (RR) were assessed by auscultation with a stethoscope at the following time points: before sedation (BS), after sedation (AS), before cervical transposition (BCT), immediately after collection (IAC), and at 0.5, 1.5, 3, 6, 12, 24, and 48 hours after collection (AC). Blood samples were collected from the jugular vein at those moments for the measurement of serum total protein, albumin, and cortisol concentrations, as well as plasma glucose levels. Blood samples were centrifuged at 1500 × g for 15 minutes, and the serum and plasma were separated and stored at -20 °C until analysis.

Serum total protein and albumin concentrations, as well as plasma glucose levels, were measured using commercial kits (Labtest Diagnóstica S.A., Minas Gerais, Brazil) in a semi-automatic biochemical analyzer (BIO 2000, Bioplus Produtos Para Laboratórios Ltda., São Paulo, Brazil). Serum cortisol concentration was determined using a commercial radioimmunoassay kit (MP Diagnostics Division, Chicago, IL, USA). The kit had a sensitivity of 0.05 ng/mL and an intra-assay coefficient of variation of 7.8%. All data collected were within the limits established by the standard curve.

### Statistical analysis

Statistical analyses were performed using a mixed model in SAS OnDemand for Academics. The model included treatment as a fixed effect, and for variables measured repeatedly over time, time and the interaction between treatment and time were also included as fixed effects. The repetition was included as a random factor, and in Experiment 2, the period was also included as a random factor. Results were considered statistically significant when P ≤ 0.05 and were interpreted as trends when 0.05 < P ≤ 0.1.

## Results

### Experiment 1

**Figure 2:**
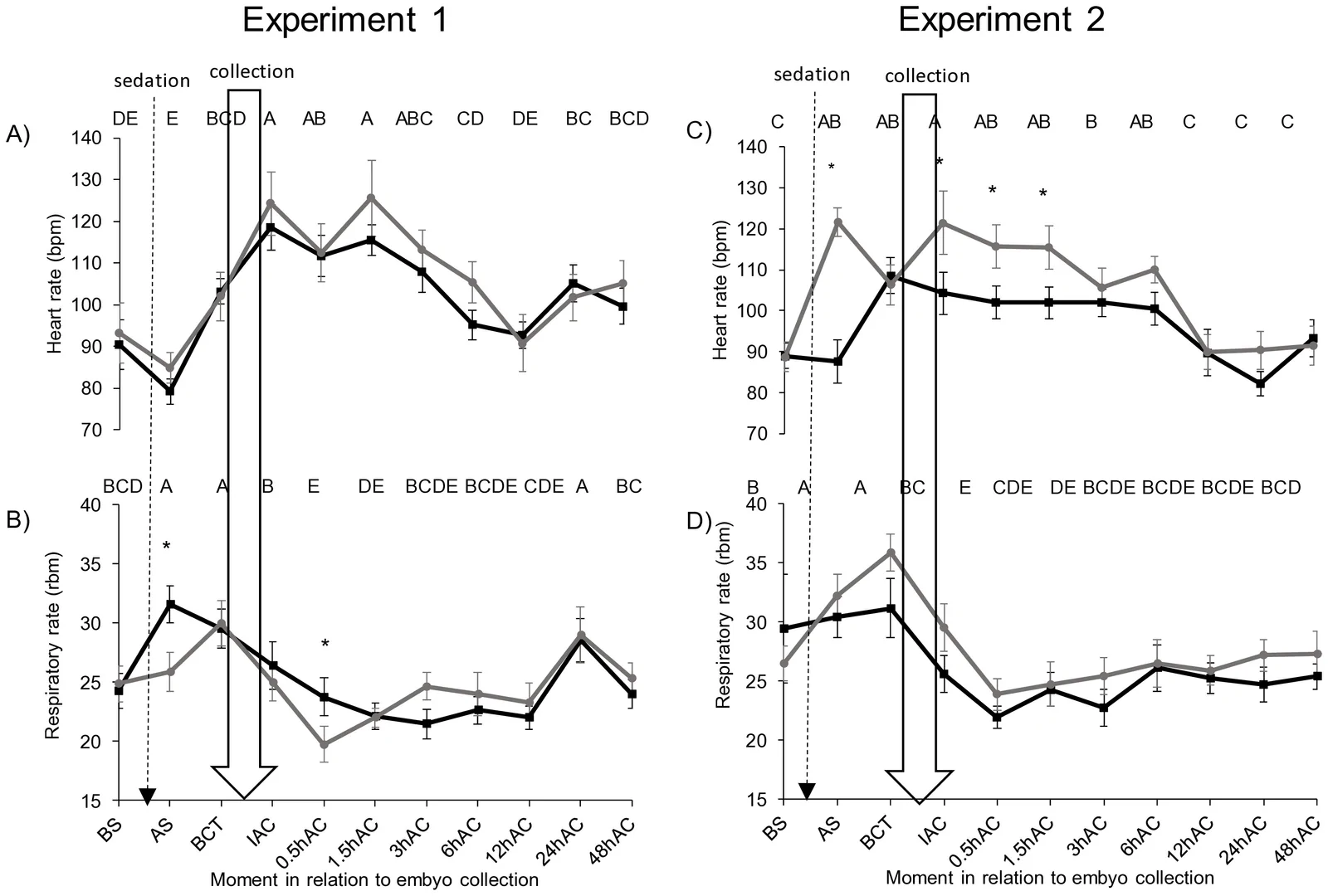
Heart rate (A, C) and respiratory rate (B, D) in Santa Inês ewes subjected to non-surgical embryo recovery. Graphs A and B refer to Experiment 1, in which animals received butorphanol and meloxicam. Graphs C and D refer to Experiment 2, in which animals received butorphanol combined with dipyrone and hyoscine. In both experiments, results from the treatment group (gray line) are compared to their respective control group (black line; saline). Data were collected at the following time points: before sedation (BS), after sedation (AS), before cervical transposition (BCT), immediately after collection (IAC), and from 0.5 to 48 hours after collection (0.5hAC to 48hAC). Vertical dashed thin arrows indicate the moment of sedation, and large arrows indicate the periods of embryo collection. Different uppercase letters indicate significant differences over time within each group (P < 0.05). Asterisks (*) indicate significant differences between groups at the same time points.

Treatments did not affect any variable, and the only interaction observed between treatment and time was in RR (P = 0.048) (Table 1). Heart rate increased from BCT to 1.5hAC, peaking at IAC and 1.5hAC (Figure 2A). Respiratory rate increased AS, BCT, and again at 24hAC. It was significantly greater in CONG1 ewes than in MBG ewes at AS and 0.5hAC (Figure 2B). Respiratory rate, HR, cortisol, and glucose concentrations varied over time (P < 0.0001 for all). The respiratory rate increased at AS and BCT, followed by a gradual decline until 12hAC. A slight rise occurred at 24hAC, after which RR declined again through 48hAC (Figure 2B).

**Table 1:** Effect of treatments, time, and their interactions on stress/pain markers in Santa Inês ewes subjected to non-surgical embryo recovery and treated with butorphanol and meloxicam (MBG) or remaining as untreated controls (CONG1). (LS mean ± SEM).

Variables	Treatments		P		
	CONG1	MBG	Treatment	Time	Interaction between treatment and time
HR (bpm)	101.7 ± 1.9	105.3 ± 2.0	ns	<0.0001	ns
RR (rpm)	25.1 ± 0.5	24.9 ± 0.5	ns	<0.0001	0.048
Glucose (mg/dL)	88.8 ± 1.9	88.1 ± 2.4	ns	<0.0001	ns
Cortisol (nmol/L)	155.8 ± 15.8	132.6 ± 11.6	ns	<0.0001	0.1
Total proteins (g/dL)	7.5 ± 0.1	7.8 ± 0.1	ns	ns	ns
Albumin (g/dL)	2.8 ± 0.1	2.9 ± 0.1	ns	ns	ns

Data are presented as means. HR: heart rate; bpm, beats per minute; RR: respiratory rate; rpm: respiratory movements per minute.

**Figure 3:**
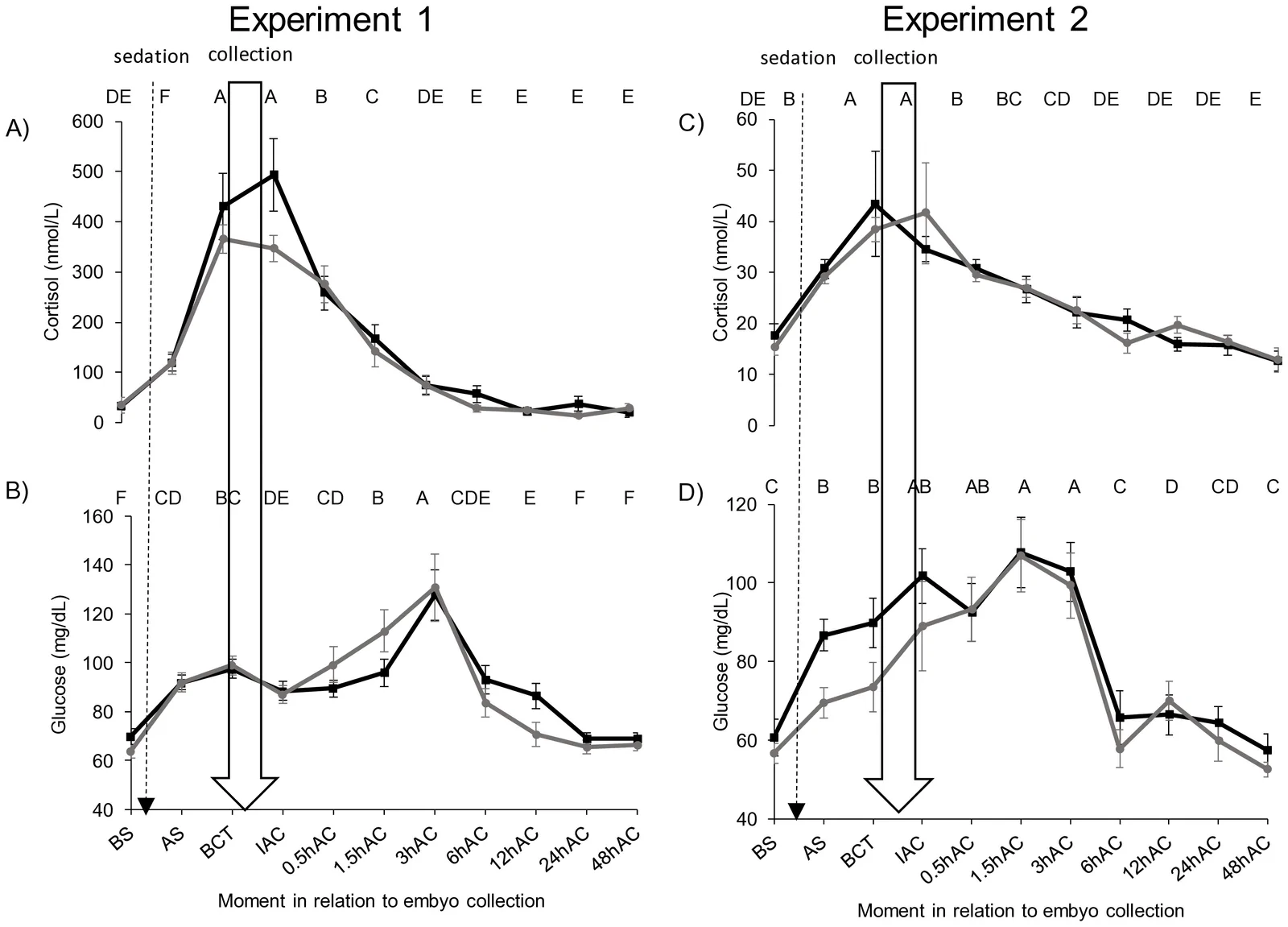
Serum cortisol (A, C) and plasma glucose (B, D) concentrations in Santa Inês ewes subjected to non-surgical embryo recovery. Graphs A and B refer to Experiment 1, in which animals received butorphanol and meloxicam. Graphs C and D refer to Experiment 2, in which animals received butorphanol combined with dipyrone and hyoscine. In both experiments, results from the treatment group (gray line) are compared to their respective control group (black line; saline). Data were collected at the following time points: before sedation (BS), after sedation (AS), before cervical transposition (BCT), immediately after collection (IAC), and from 0.5 to 48 hours after collection (0.5hAC to 48hAC). Vertical dashed thin arrows indicate the moment of sedation, and large arrows indicate the period of embryo collection. Different uppercase letters indicate significant differences over time within each group (P < 0.05).

Serum cortisol concentrations increased from BS to AS, and sharply from AS to BCT, declining gradually after IAC (Figure 3A). Although there was no overall treatment effect, a tendency for an interaction between treatment and time was observed (P = 0.1). Glycemia fluctuated during the evaluation period, peaking at 3hAC (Figure 3B). Total protein and albumin concentrations were not affected by any factor included in the model.

### Experiment 2

Heart rate was greater in BBG than in CONG2 ewes (P = 0.001), and there was also a significant interaction between treatment and time (P = 0.001). It was greater in BBG than in CONG2 ewes at AS, IAC, 0.5hAC, and 1.5hAC (Figure 2C). Respiratory rate varied only with time (P < 0.0001) (Table 2), increasing at AS and BCT, followed by a gradual decline over time (Figure 2D). Cortisol varied only with time (P < 0.0001) (Table 2), increasing from AS to IAC and declining thereafter (P < 0.0001) (Figure 3C). Glycemia was greater in CONG2 than BBG ewes (P = 0.03) (Figure 3D).

**Table 2:** Effect of treatments, time, and their interactions on stress/pain markers in Santa Inês ewes subjected to non-surgical embryo recovery and treated with butorphanol and the combination of dipyrone and hyoscine (BBG) or remaining as untreated controls (CONG2). (LS mean ± SEM).

Variables	Treatments		P		
	CONG2	BBG	Treatment	Time	Interaction between treatments and time
HR (bpm)	96.5 ± 1.4	105.2 ± 1.7	0.001	<0.0001	0.001
RR (rpm)	26.1 ± 0.6	27.8 ± 0.6	ns	<0.0001	ns
Glucose (mg/dL)	81.4 ± 2.2	75.0 ± 2.3	0.03	<0.0001	ns
Cortisol (nmol/L)	24.7 ± 1.3	24.4 ± 1.2	ns	<0.0001	ns
Total proteins (g/dL)	8.6 ± 0.1	8.8 ± 0.1	ns	ns	ns
Albumin (g/dL)	2.6 ± 0.1	2.5 ± 0.0	ns	0.04	ns

Data are presented as means. HR: heart rate; RR: respiratory rate; bpm: beats per minute; rpm: respiratory movements per minute.

The total protein concentration was not affected by the studied factors, and albumin concentration varied over time (P = 0.04). Albumin’s concentration remained stable throughout most of the evaluated periods, showing a decrease at 48hAC compared to the other moments, except for time BCT, where levels were similar.

## Discussion

Overall, based on the physiological markers assessed, the analgesic protocols tested in this study did not significantly reduce the stress responses associated with NSER in ewes under the present experimental conditions. In both experiments, the most pronounced alterations in stress indicators occurred during and immediately after the embryo recovery procedure. These findings align with previous studies, which demonstrate that, despite being less invasive than laparotomy, NSER still induces measurable physiological stress responses in ewes (Santos et al., 2020). Contrary to expectations, standard analgesic combinations were insufficient to mitigate discomfort and distress. This reinforces the importance of refining analgesic and handling protocols to enhance animal welfare during reproductive procedures. Although the combinations tested did not yield significant improvements, the data provide valuable insights into the physiological complexity of pain perception and stress modulation in sheep.

In Experiment 1, no significant main effects of treatment were observed, and only RR showed a treatment-by-time interaction, suggesting a transient effect of the butorphanol-meloxicam combination. However, RR is a highly sensitive and variable parameter influenced by numerous environmental and physiological factors; therefore, this isolated response should be interpreted with caution. The observed increases in HR, cortisol, and glucose are consistent with autonomic nervous system activation triggered by procedural stress (Moberg, 2000), indicating that animals experienced discomfort despite analgesic administration. Butorphanol has a rapid onset of action (10-20 minutes) but a short duration (2-4 hours), theoretically covering the collection period. Nevertheless, its efficacy may have been limited by suboptimal dosing, the intensity of cervical manipulation, or the influence of non-nociceptive stressors such as restraint and handling (Hemsworth et al., 2011; Weary et al., 2006). Although meloxicam exhibits a long half-life in sheep (10-15 hours) (de la Puente et al., 2024; Woodland et al., 2019), its delayed onset may render it less effective against acute nociceptive pain. Moreover, NSAIDs predominantly target inflammatory pain rather than immediate procedural discomfort (Fajt et al., 2011). These pharmacokinetic and pharmacodynamic factors may explain why the combination failed to suppress acute autonomic responses, emphasizing the need for multimodal analgesic strategies addressing both inflammatory and acute nociceptive mechanisms.

In Experiment 2, HR values were transiently higher in the treated group, likely reflecting increased sympathetic activation and catecholamine release in response to handling and procedural stimuli (Moberg, 2000). Interestingly, treated ewes exhibited lower plasma glucose concentrations compared to the control group. In ruminants, stress typically induces hyperglycemia via activation of the hypothalamic-pituitary-adrenal (HPA) axis and glucocorticoid-mediated gluconeogenesis (Bakry and Hamada, 2025; Moberg, 2000). Therefore, the lower glucose levels observed here may reflect mild sedation-related metabolic effects or drug interactions rather than reduced stress per se. Considering that basal glucose concentrations are physiologically lower in ruminants due to their reliance on volatile fatty acids (VFAs) as the main energy sources (Hamada, 1984; Kaneko et al., 2008), even small fluctuations should be interpreted within species-specific ranges (Tornquist and Rigas, 2010). Handling, restraint, and repeated sampling are known to increase cortisol and catecholamine levels, contributing to metabolic shifts during experimental manipulation (Mellor et al., 2000; Santos et al., 2020).

Cortisol elevation following NSER was consistent with previous studies in sheep (Santos et al., 2020; Ribeiro et al., 2023). The lack of significant treatment effects on cortisol concentrations suggests that neither of the analgesic combinations sufficiently attenuated HPA axis activation. Balistrieri et al. (2023) reported similar findings, with flunixin and flunixin-dipyrone combinations improving welfare indices during surgical embryo recovery in ewes, while NSAID-based monotherapy yielded limited benefits. Although the present study used a non-surgical model, the results highlight the importance of protocol refinement—potentially through adjusted dosing, additional analgesic classes, or sequential drug administration—to achieve consistent welfare improvements.

Behavioral responses are recognized as important indicators of animal welfare, particularly in response to stress and pain (Mellor and Reid, 1994; Grandin, 1997). However, in the present study, behavioral assessment was not systematically incorporated. This omission stems from the qualitative nature of behavioral observations, which can be challenging to quantify and are susceptible to observer bias, particularly in the presence of human handlers (Mellor et al., 2000). In addition to physiological and behavioral assessments, it is crucial to acknowledge the impact of human interaction during animal handling. Direct contact with the animals was necessary for repeated blood sampling and vital sign monitoring, which may itself act as a stressor (Hemsworth, 2003; Rault et al., 2020). This added stress could confound the interpretation of physiological markers by elevating stress responses unrelated to the embryo collection procedure itself. To address this, future studies could benefit from incorporating remote, non-invasive monitoring systems, such as wearable heart rate sensors, automated behavioral monitoring, or video recording, which allow for continuous observation without direct human interference (Liao et al., 2019; Rutherford, 2002). These approaches could reduce handling-induced stress and provide more accurate assessments of stress responses directly attributable to embryo collection procedures.

## Conclusion

Under the conditions of this study, the pharmacological protocols combining butorphanol with meloxicam (Experiment 1) or associating dipyrone and hyoscine (Experiment 2) did not significantly reduce the physiological stress and pain-related responses observed during NSER in ewes. Although it produced minor transient effects, these were insufficient to demonstrate consistent analgesic efficacy. The findings emphasize the need to refine pain management strategies by optimizing drug timing, dosing, and incorporating behavioral indicators to ensure animal welfare and improve the efficiency of embryo recovery procedures in sheep.

## Data Availability

Research data is only available upon request.
